# Clapping for carers in the Covid‐19 crisis: Carers' reflections in a UK survey

**DOI:** 10.1111/hsc.13474

**Published:** 2021-06-14

**Authors:** Jill Manthorpe, Steve Iliffe, Patricia Gillen, John Moriarty, John Mallett, Heike Schroder, Denise Currie, Jermaine Ravalier, Paula McFadden

**Affiliations:** ^1^ The Policy Institute at King's, King's College London London UK; ^2^ Research Department of Primary Care & Population Health Centre for Ageing Population Studies London UK; ^3^ Faculty of Life and Health Sciences School of Nursing and Institute of Nursing and Health Research Ulster University Coleraine UK; ^4^ Queen's University Belfast Belfast UK; ^5^ Ulster University Coleraine UK; ^6^ Queen's University Management School Queen's University Belfast Belfast UK; ^7^ Psychology Bath Spa University Bath UK; ^8^ Social Work Ulster University Londonderry UK

**Keywords:** carers, clapping, coronavirus, Covid‐19, survey, workforce

## Abstract

This paper reports and discusses the weekly Clapping for Carers – described as ‘front‐line heroes’ that took place across the United Kingdom during the first national lockdown of the coronavirus pandemic. Data are drawn from a UK‐wide online survey of health and social care workers, completed in May to July 2020. The survey received 3,425 responses of which 2,541 were analysed; free‐text comments were categorised. One question asked specifically: ‘Do you think the “Clap for Carers” was a helpful response from the public?’, and 815 comments were provided. Responses were extracted from these 815 free‐text comments and categorised as follows: unequivocally Yes, predominantly Yes, mixed feelings, predominantly No and unequivocally No. Most comments revealed mixed feelings about the helpfulness of Clapping with only a minority being entirely supportive. The free‐text comments offer some explanations for these views with many feeling that Clapping distracted from the severity of the pandemic and the inadequate resources. The free‐text comments reveal workforce concerns that the support demonstrated for the frontline workforce in Clapping might be transitory and that it may not translate into workforce improvements and political commitment to further funding of health and social care. Some saw the value of Clapping as illustrative of community cohesion. There was little mention of Clapping for heroes, and where it was the notion of heroism was rejected. The demonstration of public support in Clapping for Carers may have directly benefitted the public, but only indirectly the workforce. Future recruitment data may help discern if public support has translated into a desire to join the workforce.

**What is known about this topic?**

- There was strong public support for weekly public appreciation (Clapping) of carers during the first national coronavirus lockdown in the United Kingdom.
- Public appreciation of frontline workers is sometimes tempered by feelings that it is a distraction from the management of the coronavirus pandemic.
- War and heroic imagery was often used in reference to the coronavirus pandemic.

**What this paper adds?**

- The health and social care workforce had mixed views about Clapping for Carers.
- Although appreciative of Clapping for Carers, the majority expressed concerns that Clapping did not reflect tangible support.
- Many survey respondents from social care were pleased to be included in the Clapping for Carers' initiative although some felt Clapping was focused on the health service.

## 1 INTRODUCTION

Millions of people in the United Kingdom (UK) displayed their support for frontline health and social care workers in the first national lockdown by taking part in Clapping for Carers. The phenomenon occurred internationally as public recognition of the heroism of these staff (Booth et al. 2020). This paper reports data from a large UK survey of the health and social care workforce that sought respondents' feelings about this public manifestation of support and discusses the range of opinions expressed and their differentiation from those of the public.

### 1.1 Background

From its outset, experts differed on how to manage the Covid‐19 pandemic in the United Kingdom. The UK governments (England, Wales, Scotland and Northern Ireland) opted to mitigate (but not control) the effects of Covid‐19 by shielding those seen as particularly vulnerable (Brown, 2020), adopting containment measures (social distancing, working from home, and closure of public spaces and most congregate services) and restricting testing to those with symptoms. Changes in strategy had to take into account the limited personnel and resources. Before the pandemic, there were 100,000 job vacancies across the National Health Service (NHS; Rolewicz, 2020), and 7.3% of social care roles were vacant in 2019/2020, equivalent to 112,000 vacancies at any one time in England alone (Skills for Care, 2020).

A 7‐week delay in introducing containment measures was followed by recurrent shortages of Personal Protective Equipment (PPE), which provoked angry responses from NHS staff. According to the Health Service Journal (Hignett, 2020), some NHS trusts turned to alternative suppliers to source protective kit, including hardware and home decorating (DIY) shops. NHS purchasing teams reported particular difficulties in getting hold of PPE, including a lack of UK‐based manufacturers; other countries restricting exports in order to meet their local demand, poor quality products were purchased as buyers sourced items in a hurry and some unjustified price rises were introduced by suppliers. In some places, PPE was delivered to the NHS by army lorries. In adult social care, the lack of PPE was described as reflective of long‐standing concerns about standards and protection of workers and service users in these settings (Dunn et al., 2020).

Similar problems appeared when the government promised widespread screening for coronavirus cases. NHS plans to test widely were poorly executed; screening tests were not available or particularly reliable (Surkova et al., 2020) and university laboratories that could have contributed to test analysis had been mothballed and their staff sent home as part of university lockdowns (The Economist, 2020a). Overall, the government was seen by some as ‘glaringly underprepared’ for the pandemic, and in June 2020, its responses were described as ‘slow, complacent and flat‐footed’ (McKie, 2020). In the same month, The Economist judged that ‘Britain had the wrong sort of government for a pandemic … and the wrong Prime Minister’ (The Economist, 2020b, p. 7).

#### 1.1.1 Why clap?

Clap for Carers (for NHS, social care and for other key workers) was a behaviour initiated by Annemarie Plas who witnessed regular applause in The Netherlands, and who imported the idea to the UK (Rimmer, 2020). She promoted 1‐min Clapping at 8 p.m. every Thursday evening, starting on 26 March 2020, as a sign of appreciation for NHS and social care staff. The last Thursday clap was held on 28 May 2020; attempts to rekindle Clapping failed in January 2021. Other public manifestations of solidarity, gratitude and hope emerged in artworks such as murals and children's paintings of rainbows displayed in windows facing the street (BBC, 2020).

There were mixed responses to the Clapping from the public. Although joined in enthusiastically by millions, some saw it as a diversion from equipment shortages, the under‐payment of some ‘front‐line’ staff (Rimmer, 2020) or a ‘hollow act of self‐gratification’ (Wedderburn, 2020). Commenting specifically on the inclusion of social care staff, Wood and Skeggs (2020) acknowledged that this mood may be a ‘heart‐warming’ response to very real fears:

Clapping as a public form of recognition is a demonstration of our collective feeling, perhaps finally socialising some understanding of how we all ultimately rely on care workers ….

A UK survey of 1,664 adults (YouGov, 2020), conducted at the end of May 2020, reported that most (69%) had joined the Thursday night Clapping, with 29% reporting that they had done so weekly. Nearly half (44%) thought Clapping had been become politicised, although many remained supportive of it, and nearly three quarters (73%) thought that they and their neighbours were being sincere in their appreciation. Positive personal accounts of participating and its value were reported (Loveday, 2020).

Clapping for the NHS and social care, and its carers or other key workers began in March 2020, as the pandemic entered a phase in which a decisive and visible community response – by self‐isolating, maintaining social distance and attending to hygiene – was needed. In a very specific context, it contributed to the community response a collective ritual that integrated cognitive and emotional elements in praise of heroes. Brown (2020) has drawn attention to the relevance of rituals in the coronavirus period and how anthropologists have seen them as a way of diffusing conflicts and promoting inclusivity. Although public views have been explored to some extent, little is known how health and social care workers perceived the Clapping for Carers initiative at the time. In this context, the views of those who were being ‘Clapped’ that were obtained in this present study are an important addition to the story of the UK responses to Covid‐19.

## 2 METHODS

The data for this study come from the first in a series of three cross‐sectional surveys of UK health and social care workers' quality of working life and coping while working during the Covid‐19 pandemic, which launched in May 2020. The research aims to explore the impact of providing health and social care during the Covid‐19 pandemic on nurses, midwives, social care workers, social workers and patient‐facing allied health professionals (AHPs; the survey was promoted to occupations listed on the Health and Care Professions Council registrar for 2020, including occupational therapists, physiotherapists, dieticians and paramedics). These staff groups were chosen to provide a wide range of frontline practitioners in both health and social care and in different settings such as hospitals, care homes and the community. Medical practitioners were not surveyed as they appeared to be being covered by other studies such as the CoPEHCP trial (https://www.qmul.ac.uk/whri/research/cope‐hcp/).

The present article is based on data collected in the first self‐report online survey, which ran between 7 May and 3 July 2020 (the UK national lockdown started 26 March 2020 and restrictions were mainly eased on 23 June 2020). The survey was funded by several sources (see below). Necessary ethical permissions were received from the University of Ulster and information about the purpose of the survey, confidentiality and data protection were provided to potential respondents to ensure informed consent which was confirmed by survey participation. The first survey received 3,425 responses of which 2,541 were fully analysed once missing data were dealt with and are reported in the first report of the study (McFadden et al. 2020).

A purposive non‐random sampling method was adopted by the survey multidisciplinary research team, with a view to gathering information from as many willing respondents as possible and adjusting for under‐ and over‐representation where possible. Respondents were recruited through newsletters, social media posts and emails publicising the invitation to participate that were forwarded by professional regulatory bodies, associations, professional press and workplace unions for nurses, midwives, AHPs, social care workers and social workers. The survey was presented as an independent study with acknowledgement of funding support. Respondents were assured of their anonymity within the data set, and no directly identifying information was sought.

The online survey contained questions on demographic and work‐related information, validated scales assessing respondents' wellbeing, work‐related quality of life and coping strategies (see McFadden et al. 2020), with a small number of open‐ended questions enquiring about respondents' experiences of working during the early part of the Covid‐19 pandemic. One of these questions was ‘Do you think the “Clap for Carers” was a helpful response from the public?’

### 2.1 Analysis strategy

Demographic information relevant to the present paper included country and respondents' occupation (for further details of the methods and measures, see McFadden et al. 2020). Of the initial sample of 3,425 respondents, we excluded 607 who did not complete any questions past the initial demographics section; a further 272 for whom there were 30% of missing values on the relevant measures, and five respondents who did not state their occupational group (see McFadden et al., 2021). This left an effective sample size of 2,541 respondents. Given the high level of representation of respondents from Northern Ireland and of social workers in the sample, a two‐factor weighting by occupation and region was applied to all summary statistics of the sample (McFadden et al., 2021).

Analysis of the question about Clapping was undertaken by firstly analysing the response to this question using the weighted numbers and classifying helpfulness by Yes/No/Maybe categories (see Table 1), then reading any free‐text responses to the question (included under ‘Other’) and considering the reasons provided that might have contextualised or substantiated these judgements. We adopted the conventional approach to qualitative analysis of free text data advocated by Erlingsdóttir et al., (2019). Manifest content analysis, which requires researchers to concentrate on the visible and obvious meaning of texts, is an appropriate method of enquiry when participants have a stake in and experience of the topic and have the writing skills to express themselves. It allows reporting of things that might not otherwise be revealed.

**TABLE 1:** The spectrum of responses to the question: Do you think the 'Clap for Carers' was a helpful response from the public?

Unequivocal Yes 98 of 815	‘…it showed that people genuinely cared and respected the work done by carers’ (1,802, male, social worker, community) ‘It is always good to say thank you and it got people out together and connected communities. It also highlighted how under resourced the key services are’. (542, Female, social worker, community)
Predominantly Yes 31 of 815	‘…not sure how it helped the National Health Service (NHS) but it did bring our street out and talking with each other. From these weekly events we have organised socially distanced picnics and got to know each other where before we didn't before’. (2,294, Female, Community, social worker)
Mixed feelings 381 of 815	‘Frontline staff needed to know that their commitments to the service were acknowledge by millions of people to boost the morale because whilst staff themselves were at risk leaving their family behind to do their job. However [there were] people who did not clap for carers because they were very angry seeing the scene when NHS has been long underfunded, staff under‐paid for the job they do’. (670, Female, community, nurse)
Predominantly No 137 of 815	‘As worthy as the NHS is for the “clap for carers” movement, I was increasingly frustrated at the lack of acknowledgement for care staff in domiciliary care, supported living, or care homes. Continually seeing discounts offered to NHS staff, but not to care staff who were working just as hard. It was a positive moment for the community, but also somewhat alienating’. (918, Female, day care, social care worker)
Unequivocal No 168 of 815	‘It distracts from the issue that Health and social care are underfunded, and a more useful response would be a campaign of lobbying government to provide adequate funding. It will not, I believe, make a long‐term difference to social care and health policy’. (812, Male, Community, day care)

A total of 815 people responded to the question about Clapping by making a free‐text comment. Five categories emerged from our reading and classification of these comments, reflecting different dimensions of helpfulness beyond simple ‘yes/no/maybe’. We developed two gradations of ‘helpfulness’ or otherwise – ‘predominantly’ and ‘unequivocally’. A fifth category of ‘mixed’ was chosen to reflect opinions about Clapping, which could not be classified as whether the respondent viewed it simply helpful or not.

## 3 FINDINGS

### 3.1 Respondents’ opinions on the helpfulness of ‘Clapping for Carers’

**FIGURE 1:**
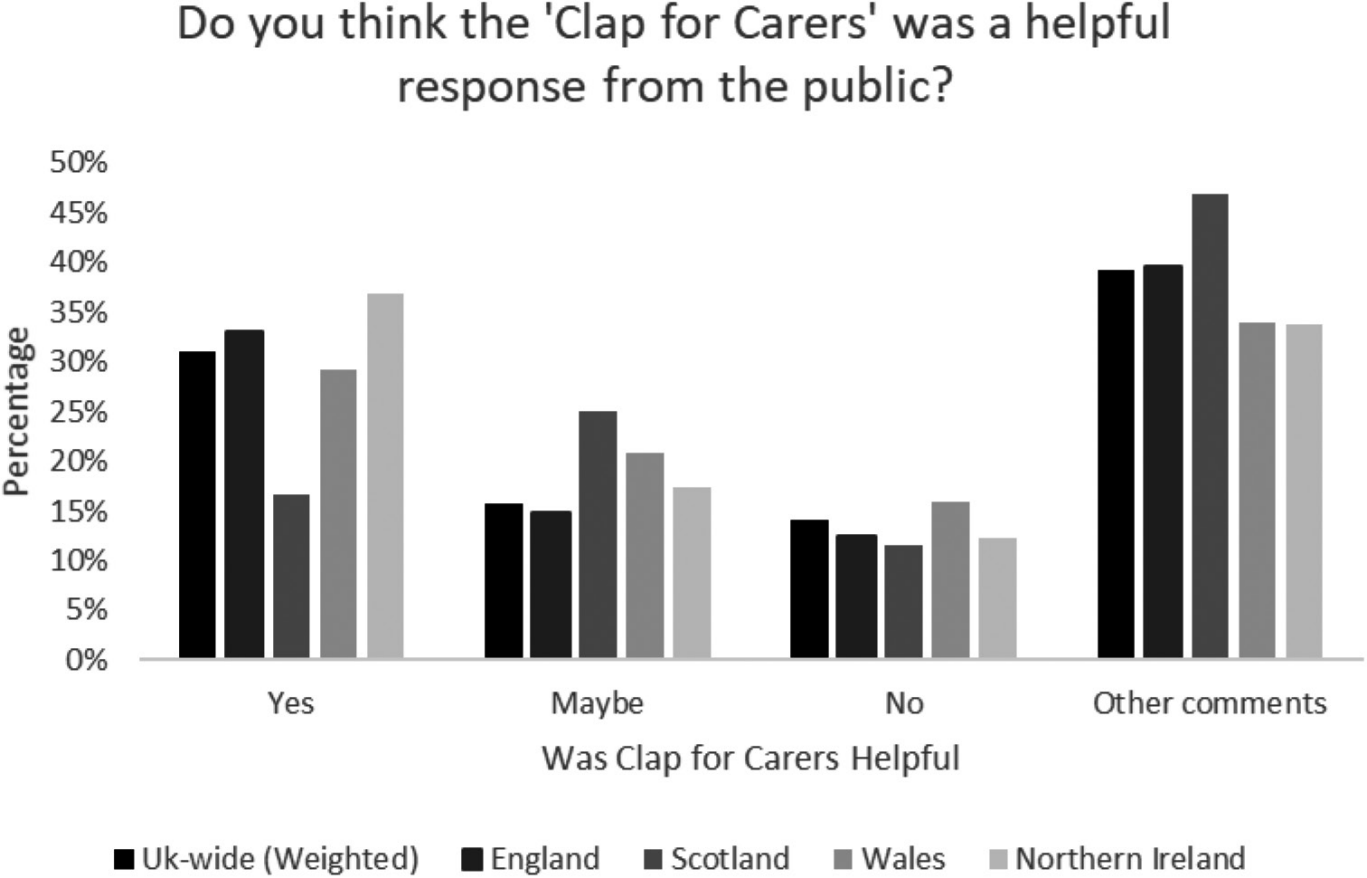
Opinion of ‘Clap for Carers’ by country

**FIGURE 2:**
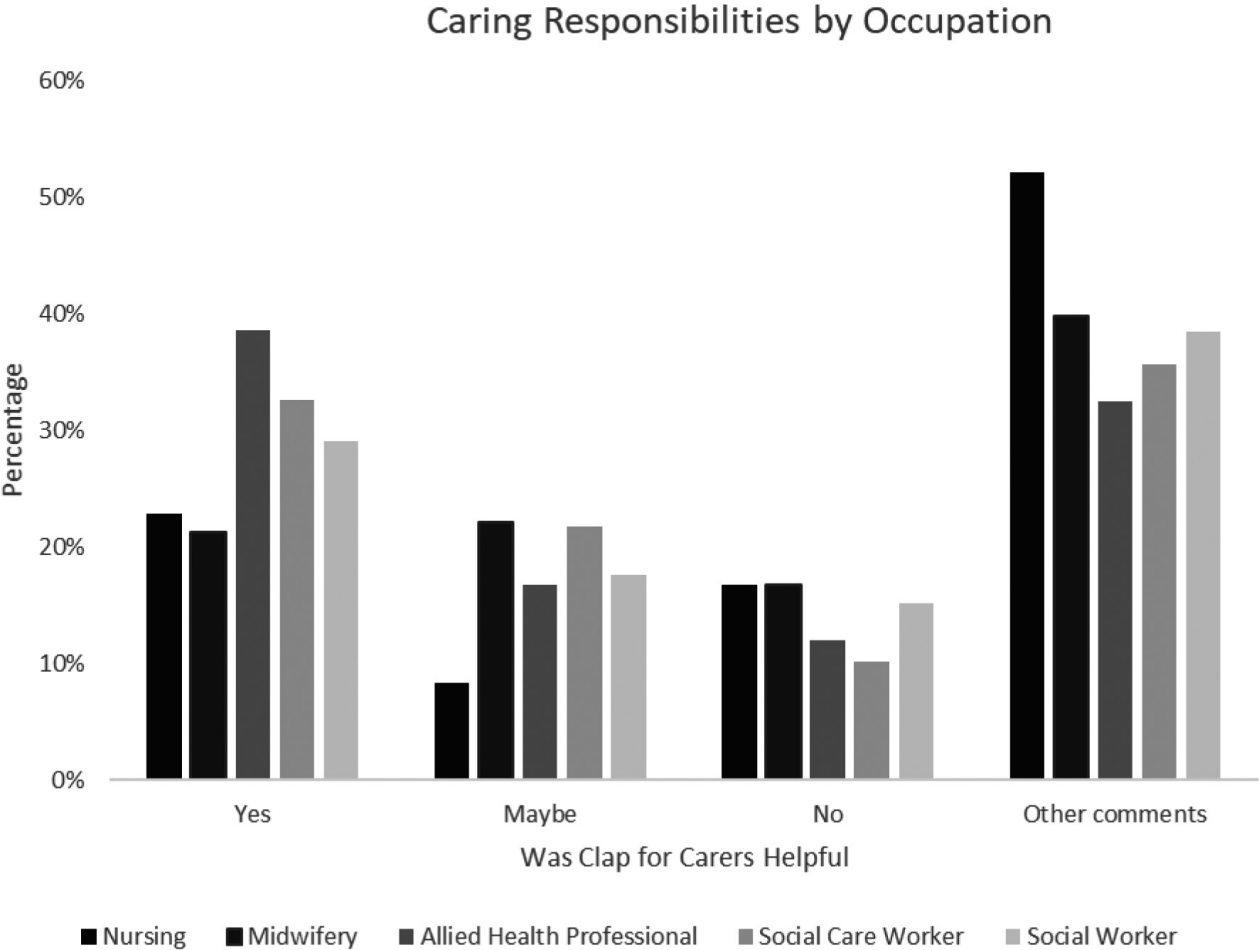
Opinion of ‘Clap for Carers’ by occupation

Around one third of respondents overall considered Clapping for Carers a helpful response, whereas 14% thought it was not. Weighted data by country revealed some differences (respondents from Scotland were least likely to see it as helpful and most likely to give a more cautious ‘maybe’; see Figure 1). These differences were also reflected by occupation (see Figure 2). Midwives and nurses were most likely to perceive it negatively.

Of the 815 free‐text comments analysed, we illustrate each of five categories with selected quotations. Analysis of the comments revealed greater granularity of the categories than found in the quantitative statistics reported above. Overall the free‐text comments expressed more mixed feelings about helpfulness, 381 out of 815, although overall there were higher numbers for what we categorised as unequivocally No (168) and predominantly No (137), compared with predominantly Yes (31) and unequivocally Yes (98). Comments were classified as ‘unequivocal’ when all elements of the comment were in agreement; see, for example, Care home worker 338, below, who refers to positive effects on both the community and on individuals. Comments that were ‘predominantly’ for or against clapping had more components of the comment in one direction than the other. Such statements often contained words like ‘however’ or ‘but’ (see Care home worker 4, below). Uncertainties about classification of comments were resolved through discussion within the research team.

The smallest number of comments was unequivocal in their views that Clapping for Carers was helpful: both reflecting a feeling of appreciation but notably in helping promote and give voice to a sense of community and to bring communities together. If social distancing was maintained, it was helpful in sustaining community cohesion, and from this, more tangible mutual community support may emerge or be sustained. As one care home worker observed, it was ‘Good for community morale – people stepped out of their front doors at a time when everything else was indoors’ (Care home worker 338). Few commented on the positive impact on themselves personally, although some who did made reference to feeling appreciated, albeit with some caveat: ‘It was a lovely sentiment and appreciated. However, what would be more appreciated is increased pay/fair pay, better communication, respect for NHS services and social care’ (Care home worker 4).

Whereas the illustrative quotes of each of the categories contained in Table 1 (below) are mainly from social care workers, the majority participant group, they were also voiced by other respondents.

At the second level of coding of the free‐text comments, a variety of subthemes was found, often cutting across categories. For example, Clapping could be seen as predominantly a positive initiative that was well intentioned and the reasons that apparently lay behind the reservation about it could be that it went on for too long or was being used by politicians as a diversion from underfunding of health and social care. An increase in pay, better funding for the NHS and social care, with recognition and appreciation for health and social care staff on an ongoing basis, were cited in many of the free‐text comments as being what were really needed.

However, some did see it as a welcome personal or public recognition of their work and their service: ‘I think it's been helpful for the NHS to keep their spirits up and allow them to reflect on the crucial role they are playing and see pride in that’ (Social care worker in the community, 709). Such a focus on the NHS did not always meet with approval and explained some of the reservations lying behind predominately positive or negative judgements or mixed views. For example, some doubted whether social care staff were being included and so were seen as having mixed views not necessarily being critical about Clapping but who was being included: ‘As worthy as the NHS is for the 'clap for carers' movement, I was increasingly frustrated at the lack of acknowledgement for care staff in domiciliary care, supported living, or care homes. Continually seeing discounts offered to NHS staff, but not to care staff who were working just as hard. It was a positive moment for the community, but also somewhat alienating’ (Social care worker working in supported living service 22).

Others unequivocally considered Clapping patronising, but more seldom, it was viewed contradictory because those clapping might break lockdown rules, thereby putting staff at risk: ‘There was no point on a lot of individuals clapping on a Thursday night for staff and then continuing to flout rules and continue risking the people they were clapping for’ (Nurse, band 4, 737). Those free‐text comments that were unequivocally negative about Clapping regarded it as diversionary and politically motivated, as illustrated in this comment: (it is) ‘Completely futile and a political stunt designed to shift focus away from chronic underfunding and poor handling of the pandemic’ (AHP 36).

Only 22 of the 815 respondents who added free text opinions mentioned heroes or heroism which had sometimes been associated with the expressions of support for frontline workers in media reports. Among these, most rejected the idea of heroism and saw proclamations of heroism as false and designed to avoid criticism for poor treatment of health and social care staff. The few respondents who were more positive about hero status pointed out how unequally distributed it was. Typical of the rejection of hero status were a nurse and a social care worker who stated:

I also disagreed with the “hero worship” idea that went along with the clap – we're not heroes, we're professionals doing a job – calling us heroes just makes other people feel better when we die. (Nurse 145)

…front line staff are not heroes, they are people doing what they are trained to do. (Social care worker 169)

A few viewed any association between Clapping and heroism as particularly inappropriate or exclusive; the majority of such comments were from social care and social work staff:

NHS staff were the focus, and I think even they became uncomfortable with the hero status. (Social care worker 296)

I feel it put excessive pressure on NHS STAFF TO BE 'HEROES'. THIS WAS A VERY FRIGHTENING TIME. (Social worker 574 – capitals in original)

Being NHS staff should not require one to be a self‐sacrificing hero, and I doubt any of the staff wanted to have to be that. (Social worker 685)

## 4 DISCUSSION

### 4.1 Strengths and limitations

This study is limited in that survey respondents were self‐selecting and may have had particular reasons for completing the survey or for completing the free‐text options. Analysis of the survey findings was assisted by large numbers and little missing data, but analysis of free‐text comments was more limited as most free‐text comments were brief, and this did not permit more thematic exploration. The study was independent of government, employers or professional regulators and associations, and this may have encouraged frank responses. Respondents came from a variety of front‐line occupations and locations, which both limits the specificity of our findings but adds substantial breadth. However, medical practitioners were not included in the survey and their perceptions of Clapping may differ. From a surgical perspective, Moura (2020), for example, reported that participants saw it as an expression of solidarity. Findings from other early, small workforce interview‐based studies in England (e.g. Aughterson et al., 2021; Nyashanu et al., 2020) reflect some of the present study's findings about mixed feelings of being on the frontline, as does a Skills for Health UK survey (Enbank, 2020) whose 2,363 respondents spanned front‐line staff, senior managers and HR professionals from across health and social care settings and like the present study highlighted human resource requirements and developments. Other research has focused on specific sectors or nations, with the Institute for Employment Studies (Griffin, 2020, p. 6) concluding that the adult social care workforce in England considered itself to be playing a critical role and working under extraordinary pressures in response to the Covid‐19 pandemic yet felt it remained poorly understood. A range of other studies is emerging covering specialisms, such as Speech and Language Therapists working in Ear Nose and Throat services (Patterson et al., 2020), that permit specific clinical foci addressing Covid‐19 related pressures at work. Although the focus of this present study is UK wide, opportunities will emerge for international comparisons in health services work (e.g. with health workforce surveys from Spain, Mira et al., 2020 and Canada, Smith et al., 2021) and further international reflections on the Clapping phenomenon, which took place in many other countries (Booth et al., 2020).

Our findings on Clapping for Carers in the United Kingdom from the perspectives of the intended beneficiaries will inform these and wider debates about public support and its impact on the frontline of care. We found that practitioner views mirrored to some extent those of the public in welcoming this demonstration of public support, but large numbers of practitioners were slightly more cynical about the diversionary potential of Clapping from the reality of problems being experienced. And there were many who did not find it supportive. Although not critical of the public, they feared it was being manipulated or seduced by positive affirmations that would not call politicians to account. Such patterns of thought were not so commonly expressed in public surveys, but even among the public, there were some doubts about Clapping. For the public, Clapping may have served other purposes of community cohesion and survey respondents, who of course are local neighbours and friends, sometimes acknowledged this. Clapping therefore may have been an expression of some public sense of relief about surviving with people also clapping for themselves and their networks and providing a supportive ritual in uncertain times (McCormick, 2020).

Questions remain of whether there will be a lasting effect from Clapping and how this memory may influence attitudes and behaviours towards frontline work in health and social care. There are several possibilities here, including whether politicians will feel that support for the workforce needs to be visible and expressed in firm commitments [as suggested in the NHS England (2020) People Plan] and how workforce stakeholders are using memories of Clapping and intimations of heroism as indications of public support for their claims. The Head of the Royal College on Nursing, for example, called for ‘No medals, badges or claps this time – just pay nursing staff fairly’ (Kinnair, 2020). Recruitment studies may also determine if the memory of Clapping appears to act as motivating factors for jobs and professional training, as suggested by some, ‘Clap for carers' generation sets sights on career in nursing’ (Griffiths & Meddings, 2020).

Our survey spanned both health (excluding medicine) and social care, with social care including social work staff responding in large numbers. Although noting that some were equivocal about Clapping, several social care staff made favourable mention of being included as frontline staff worthy of appreciation directly or indirectly seen positively by association. This may contrast with the general lack of public valuing of social care staff whose work is often seen as low status and needing only low‐level skills (Moriarty et al., 2018).

## 5 CONCLUSION

This large survey asked a timely question about Clapping for Carers in the UK context of the Covid‐19 pandemic and its first national lockdown. Reponses exposed the possibility that the Clappers may have benefitted more than the Clapped for, but in the longer term, these demonstrations of support may serve as a reminder to politicians that the health and social care workforce matters and that working conditions and pay for these groups need to be reflective of the important role they have in society. For social care occupations in particular, the Clapping for Carers campaign might have helped to draw attention to this occupational group as well as to social care work, which has long been undervalued by society relative to other health and social occupations (Cooke & Bartram, 2015).

Speculations about the direct or indirect impacts on workforce recruitment and retention may need to be addressed by longer‐term studies.

## CONFLICT OF INTEREST

The authors declare that they have no conflict of interest and that they satisfy the conditions for authorship.

## AUTHOR CONTRIBUTION

The list of authors accurately illustrates who contributed to the work and how. All those listed as authors qualify for authorship.

## Data Availability

The data that support the findings of this study are available from Dr Paula McFadden (p.mcfadden@ulster.ac.uk) upon reasonable request.
